# Eisenmenger Syndrome in Pregnancy

**DOI:** 10.5935/1678-9741.20160062

**Published:** 2016

**Authors:** Shi-Min Yuan

**Affiliations:** The First Hospital of Putian, Teaching Hospital, Fujian Medical University, Fujian Province, China.

**Keywords:** Eisenmenger Complex, Pregnancy Complications, Hypertension, Pulmonary

## Abstract

Eisenmenger syndrome is very rare in pregnant women. Debates remain concerning the management of Eisenmenger syndrome in this patient population and the prognosis is unclear in terms of maternal and fetoneonatal outcomes. Epidural analgesia is preferred for Cesarean section as it alleviates perioperative pain and reduces the pulmonary and systemic vascular resistances. Maternal mortality in the presence of Eisenmenger syndrome is reported as 30-50% and even up to 65% in those with Cesarean section. The major causes of death could be hypovolemia, thromboembolism and preeclampsia. Pregnancy should ideally be avoided in a woman with Eisenmenger syndrome concerning the high maternal mortality rate and probable poor prognosis of the baby. A short labour and an atraumatic delivery under epidural block are preferred in the women with a strong desire of pregnancy. The purpose of this article is to discuss the debates of Eisenmenger syndrome in pregnancy and the possible resolutions.

**Table:** 

Abbreviations, acronyms & symbols	
PAH	= Pulmonary artery hypertension
PVR	= Pulmonary vascular resistance
SVR	= Systemic vascular resistance

## INTRODUCTION

Pulmonary artery hypertension (PAH) is a devastating and refractory disease^[1]^. It is rarely reported in pregnant women, but it is associated with significant morbidity and mortality of both mother and baby^[2]^. In 1897, Victor Eisenmenger described a large ventricular septal defect as well as the pathological features of PAH of a 32-year-old man and therefore the condition was termed as Eisenmenger syndrome^[3]^. In 1958, Wood^[4]^ expounded this syndrome as a result of an increased pulmonary vascular resistance (PVR) > 800 dynes/sec/cm^-5^ with a reversed or bidirectional shunt through a large ventricular septal defect. Eisenmenger syndrome is very rare in pregnant women with an incidence of about 3% in the pregnant patients with congenital heart defects^[5]^. Nevertheless, debates remain concerning the management of Eisenmenger syndrome in this patient population and the prognosis is unclear in terms of maternal and fetoneonatal outcomes. The aim of this article is to discuss the debates of Eisenmenger syndrome in pregnancy and the possible resolutions. The study materials stem from a comprehensive retrieval literature of 1970 to present with search terms of Einsenmenger syndrome and pregnancy.

## CLINICAL MANIFESTATION

In pregnant women, the congenital heart diseases that cause pulmonary vascular disease and evolve into Eisenmenger syndrome are mainly ventricular septal defect, followed by atrial septal defect and patent ductus arteriosus^[6]^. The pregnant women with Eisenmenger syndrome may present with cyanosis or differential cyanosis, dyspnea, fatigue, dizziness and even right heart failure^[6]^. Physical examinations may reveal cyanosis and clubbing of the fingers^[7]^. Hemorrhagic tendency, such as epistaxis and hemoptysis, has been reported^[8]^. Auscultation may reveal an inspiratory crepitation^[9]^ and a loud P_2_ and a systolic murmur at the pulmonary area. Jugular venous distention and mild lower extremity edema can be seen^[7]^. Once the patients develops Eisenmenger syndrome, the machinery murmur might be unaudible and the associated patent ductus arteriosus might be misdiagnosed^[10]^. Patients may have a low oxygen saturation^[11]^ and polycythemia^[12]^. Severe complications, such as heart failure, endocarditis and thromboembolic accidents, may develop in the condition of pregnancy. Delivery by a pregnant woman with Eisenmenger syndrome represents an increased risk of pulmonary thromboembolism and sudden death, often occurring within the first few days of postpartum^[11]^. A chest X-ray may reveal cardiomegaly with bilateral pulmonary congestion^[9]^. Electrocardiogram demonstrates right ventricular hypertrophy and sometimes left ventricular hypertrophy. Cardiac catheterization can be used to locate the defect and detect pulmonary arterial pressure^[13]^.

## PATHOPHYSIOLOGY

**Fig. 1:**
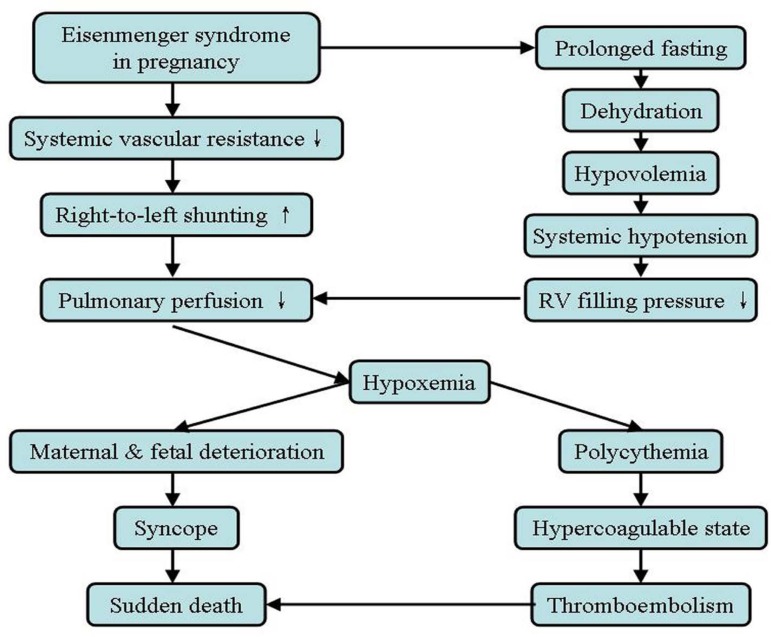
Pathophysiology of Eisenmenger syndrome in pregnancy^[7,13,16]^. RV: right ventricle.

The main pathophysiological changes can be cyanosis due to a series of hematological and hemodynamic disorders, including secondary erythrocytosis, increased blood viscosity, iron deficiency anemia, blood clotting disturbances, heart failure and serious rapid arrhythmias^[14]^. Eisenmenger syndrome patients are particularly vulnerable to hemodynamic changes induced by anesthesia or surgery, and even minor decrease in systemic vascular resistance (SVR) may increase the right-to-left shunting and possibly induce circulatory collapse. Additional risks of surgery include excessive bleeding, postoperative arrhythmia, deep vein thrombosis and paradoxical emboli^[15]^. The decreased SVR during pregnancy increases the right-to-left shunting, subsequently leading to a reduced pulmonary perfusion and hypoxia and further deterioration of mother and baby^[8]^. Figure 1 depicts the pathophysiology of the pregnant patients with Eisenmenger syndrome^[7,13,16]^. Moreover, straining during delivery may result in an increased right ventricular pressure, which may cause fatal arrhythmia and even sudden death^[13]^. Microvascular injury stimulates production of growth factors and enzymes, which causes intimal proliferation, medial hypertrophy in association with endothelial dysfunction and platelet adhesion, and leads to obliteration of pulmonary vasculature^[17]^.

## ANESTHESIA

The anesthesia for patients with PAH and mode of delivery is controversial. During labor, uterine contraction causes autotransfusion and may increase cardiac output by 25%. This increases pulmonary arterial pressure and may precipitate heart failure or arrhythmia. Regional anesthesia is potentially risky because it may decrease SVR, which would increase the shunt and exacerbate hypoxemia^[18]^. When epidural analgesia was chosen for perioperative pain, it reduces PVR and SVR by sympathetic block and reduces catecholamine levels, thus causing less tachycardia, less myocardial oxygen consumption and reduction of the right-to-left shunting^[19]^. Boukhris et al.^[18]^ successfully used epidural anesthesia in a pregnant woman with a single ventricle and Eisenmenger syndrome and provided excellent analgesia. General anaesthesia can lower SVR remarkably thereby worsening the right-to-left shunting and leading to a difficult extubation. Cole et al.^[20]^ attempted incremental spinal anesthesia using spinal catheters for elective Cesarean section in a patient with Eisenmenger syndrome and obtained a satisfactory anesthetic effect. By a graded spinal block, quicker motor block can be achieved with a reduced requirement of anesthetics in comparison to epidural anesthesia^[21]^. Table 1 shows a comparison of the anesthetic risks in pregnant patients with Eisenmenger syndrome. Of all anesthetic techniques, epidural and incremental spinal anesthesias are the only praised ones.

**Table 1:** Risks of anesthesia in pregnant patients with Eisenmenger syndrome[18-20,22].

Anesthesia	Risk
Regional	1. decreases systemic vascular resistance; 2. increases right-to-left shunting; 3. exacerbates hypoxemia
Lumbar	1. potential spinal hematoma
Epidural	1. none declared
General	1. decreases venous return and cardiac output; 2. lowers systemic vascular resistance; 3. worsens right-to-left shunting; 4. worsens oxygen saturation; 5. difficult extubation
Incremental spinal	1. none declared

Parturition and puerperium are the most hazardous moments of pregnant patients with Eisenmenger syndrome. The mode of delivery is a topic of debate. Cesarean section should be avoided due to the potentially fatal decrease of circulating blood volume in patients with Eisenmenger syndrome; while vaginal delivery appears to be safer^[23]^. Epidural anesthesia reduces the chance of precipitous hemodynamic changes, and is safe to administer epidural anesthesia to patients with Eisenmenger syndrome^[24]^. Epidural or intrathecal morphine sulphate can be devoid of effect on systemic blood pressure and represents the best approach to anesthetic management in such patients^[16]^. In general, early induction with a short labour and an atraumatic delivery under epidural block is preferred^[23]^.

## TREATMENT

Women with Eisenmenger syndrome are advised to avoid pregnancy, or an early pregnant interruption should be within 10^th^ gestational week, or with tubal sterilization or artificial abortion^[8]^. When the patient has a strong desire of pregnancy, a multidisciplinary consultation is necessary^[8]^.

Management of patients with Eisenmenger syndrome includes oxygen therapy and the use of digitalis, diuretics, vasodilators and anticoagulants^[7]^. Facemask oxygen can improve patients' hypoxic condition and reduces pulmonary artery pressure^[21]^. However, in patients with Eisenmenger syndrome, oxygen is a pulmonary vasodilator, which decreases the blood flow across the right-to-left shunt and thereby improving oxygen saturation. Maternal arterial oxygen tension should be kept at ≥70 mmHg when possible. The use of digitalis with diuretics should be cautious concerning the increased risk of digitalis toxicity. Diuretics may be useful for patients with Eisenmenger syndrome and severe right heart failure in order to relieve hepatic congestion or increase intravascular volume^[25]^. In anticipated preterm birth baby, antenatal steroids for fetal lung maturity is warranted^[7,9]^.

However, debates remain in the prophylactic anticoagulant therapy in the peripartum period. Admittedly, heparin use may prevent thromboembolic complications, but subsequent bleeding has been reported with significant blood loss and transient blood pressure drop that warrants cessation of heparin and aggressive transfusion in postpartum^[11]^. Pitts et al.^[26]^ emphasized the potential of excessive bleeding during the postoperative or postpartum period as a result of heparin therapy as a cause of death. Fang et al.^[7]^ suggested anticoagulant use should be cautious in such patients with a high risk of hemorrhage with thrombocytopenia and coagulopathy.

Use of inhaled nitric oxide during labor has been recommended in patients with Eisenmenger syndrome. Nitric oxide inhalation during the labor in the pregnant woman with Eisenmenger syndrome may improve oxygenation and attenuate pulmonary arterial pressure^[18,27]^. Inhaled nitric oxide also has an antithrombotic effect^[28]^. In a pregnant woman with severe PAH, her condition worsened more rapidly despite maximal oxygen therapy on postpartum day 3, showing severe hypoxemia. When the oxygen saturation could not be maintained above 60%, the patient was intubated. Oxygen saturation remained low until nitric oxide was given via the endotracheal tube, and an immediate improvement of oxygen saturation and hemodynamics were achieved by titrating up nitric oxide concentration to 80 ppm^[29]^. Kandasamy et al.^[22]^ avoided the use of nitric oxide because it is a potent pulmonary vasoconstrictor. Instead, they used sevoflurane because the effect of this drug on SVR may be reversed more quickly than isoflurane or halothane.

Mishra et al.^[24]^ reported antenatally irregular treatment with tablet sildenafil 25 mg twice a day in pregnant patients with Eisenmenger syndrome. Lacassie et al.^[30]^ reported a woman with severe PAH due to Eisenmenger syndrome treated during pregnancy, delivery and postpartum with sildenafil 150 mg/day along with L-arginine 3 g/day and facemask nitric oxide 64 ppm, leading to a significant reduction of PAH and PVR and clinical improvement within short time. Clinical observations revealed that PAH specific therapies (prostanoids, endothelin receptor antagonists and phosphodiesterase-5 inhibitors, single or in combination) had a significantly lower risk of death over a median follow-up of 4 years^[31]^. Cartago et al.^[32]^ reported two cases of Eisenmenger syndrome patients treated with sildenafil as monotherapy caused stabilization of the maternal condition and good clinical outcome. Prostaglandin E_1_ nebulization helps to reduce intracardiac shunting flow, improves hypoxia and decreases pulmonary artery pressure^[21]^. In spite of the already defined safe limit for surgical repair of the heart defect for patients with PAH, such as 6 Wood Unit of PVR, pulmonary to systemic flow ratio >1.5 and PVR < SVR^[33]^, cardiac operation is preserved for the pregnant patients.

## PROGNOSIS

The greatest risk lies in the periods of delivery and early postpartum due to large hemodynamic changes^[11]^. The major causes of death could be hypovolemia, thromboembolism and preeclampsia^[8]^. Alternative causes of death are massive hemoptysis, subarachnoid bleeding, heart failure, arrhythmias, cerebral abscess, complications of cardiac or non-cardiac surgery, or consequences of exercise and pregnancy^[9,19]^. Cesarean sections and other operations were associated with an even higher maternal mortality rate^[8]^.

Pregnant patients with Eisenmenger syndrome complicated by severe preeclampsia showed an extremely high mortality. Despite vaginal delivery is a preferred delivery mode, Cesarean section has been an option for many situations, such as severe intrauterine growth retardation^[22]^. Kansaria & Salvi^[16]^ reported a case of Eisenmenger syndrome in pregnancy and the patient died three weeks postpartum after a term vaginal delivery. Phupong et al.^[34]^ reported a 30-week pregnant woman with Eisenmenger syndrome and severe preeclampsia in whom Cesarean section was performed due to severe preclampsia and an unfavorable cervix under general anesthesia. The patient died of pulmonary embolism on the postoperative day 2.

Maternal mortality in the presence of Eisenmenger syndrome is reported to be 30-50%^[35]^ and even up to 65% in those with Cesarean section^[36]^. Yentis et al.^[37]^ reported that in the pregnant patients with Eisenmenger syndrome the maternal mortality was 40% and fetal demise was 8%, and only 15% of infants were born at term. Gleicher et al.^[38]^ reported a 34% mortality associated with vaginal delivery and a 75% mortality associated with Cesarean section. It is reported that the most dangerous period is early after delivery, with 70% of deaths occurring on postpartum days 2-30 or died just at the time of delivery^[39]^. Avila et al.^[35]^ studied 13 pregnancies in 12 women with Eisenmenger syndrome continuing their pregnancy. Cesarean section was performed in all patients as a result of worsening maternal or fetal clinical condition during the third trimester of gestation. Only one of them died on the 30^th^ postpartum day and only one baby died after birth.

Poor prognostic signs in maternal congenital heart disease include maternal hematocrit >60%, arterial oxygen saturation <80%, right ventricular hypertension and syncopal attacks. A fixed PAH not responsive to oxygen also carries a grave prognosis and may be an absolute indication to terminate the pregnancy^[22]^. The prognosis of such patients depends on the nature of PAH, with estimated maternal and fetoneonatal mortality of 28% and 7%, respectively^[36]^. Maternal mortality showed disparities depending on the associated congenital heart defects: it was higher when associated with ventricular septal defect than with atrial septal defect or with patent ductus arteriosus (60% *vs*. 44% *vs*. 41.7%)^[9]^. The majority of maternal deaths occurred during or within the first week after delivery. Only 25.6% of all pregnancies reached term. At least 54.9% of all deliveries occurred prematurely^[39]^. Neither the mode and timing of delivery nor the type of anesthesia and monitoring correlated with maternal outcome^[18]^. A delayed diagnosis, a delayed presentation to hospital and severity of PAH have been found to be contributing risk factors^[18]^. Cesarean sections and other operations are associated with extremely high maternal mortality during pregnancy^[38]^. In addition, hemorrhage secondary to prophylactic anticoagulation can be fatal in pregnant patients^[26]^.

A premature delivery occurred in 86% of the women and intrauterine growth retardation was 24%^[40]^. The degree of maternal hypoxemia is the most important predictor of fetal outcome as it has been noted that prepregnant arterial oxygen saturation is in a direct proportion to live births^[41]^. Vaginal delivery was associated with a 34%, Cesarean section, a 75%, and pregnancy interruptions, a 7% mortality^[38]^. Furthermore, the fetal outcome among mothers with cyanotic heart disease correlates well with maternal hematocrit^[23]^. Successful pregnancy is unlikely with a hematocrit >65%, and over 30% of the fetuses have growth retardation^[16]^. Daliento et al.^[3]^ found a high incidence of spontaneous abortions (35.8%) and cardiac abnormalities in offspring (20%) in association with maternal Eisenmenger syndrome. Prolonged bed rest, use of heparin, and oxygen therapy can produce satisfactory maternal and fetal outcomes^[22]^.

## CONCLUSION

Pregnancy should ideally be avoided in a woman with Eisenmenger syndrome because of a high maternal mortality rate and probable poor prognosis of baby. These patients with continuing pregnancy should be assessed by a combined and experienced multidisciplinary team, including obstetric, anesthetic, cardiology, pediatric and neonatal physicians. Epidural anesthesia is preferred in Cesarean section. A short labour and an atraumatic delivery under epidural block are preferred.

**Table:** 

Authors’ roles & responsibilities	
SMY	Study conception and design; analysis and/or interpretation of data; manuscript writing; final approval of the manuscript
